# Implementation of self-directed learning within clinical clerkships 

**DOI:** 10.3205/zma001439

**Published:** 2021-02-15

**Authors:** Navina Röcker, Christian Lottspeich, Leah T. Braun, Benedikt Lenzer, Jessica Frey, Martin R. Fischer, Ralf Schmidmaier

**Affiliations:** 1 Klinikum der Universität München, Medizinische Klinik und Poliklinik IV, Munich, Germany; 2 LMU München, Klinikum der Universität München, Institut für Didaktik und Ausbildungsforschung in der Medizin, Munich, Germany; 3 Charité – Universitätsmedizin Berlin, Klinische Chemie und Pathobiochemie, Institut für Laboratoriumsmedizin, Berlin, Germany

**Keywords:** curricular development, clinical clerkship, learning objective, self-directed learning, undergraduate medical education

## Abstract

**Background: **The main aim of medical curricula is to prepare students for the first day at the work place. While teaching clinical competence is pivotal, clinical clerkships are often the last chance to close knowledge gaps with the help of clinical teachers. Self-directed learning is a dynamic field for research within medical education, though its curricular implementation is rare. This study focuses on the needs assessment of clinical clerkships using the concept of self-directed learning.

**Methods: **The study comprised an educational experience at the Ludwig-Maximilians Universität (LMU) Munich. Medical students (n=1446, 59% female) in their second clinical year were instructed to specify learning objectives (LOs) by Doran`s SMART criteria and to gauge the probability of their fulfilment prior to the mandatory clerkship. In a second questionnaire one week later, the students rated the actual subjective fulfilment of the LOs. Data was coded with regards to the German National Catalogue of Competence-Based Learning Objectives for Undergraduate Medical Education (NKLM) and investigated qualitatively. Factors that determine goal achievement were collected and coded binary (barrier vs. enabler). Univariate analysis was used when appropriate.

**Results: **The acquisition of “clinically practical abilities” (29%), “diagnostic methods” (21%) and “professional communication” (13%) were the LOs mentioned most. Throughout the week, subjective fulfilment diminished. Rich (vs. poor) availability to “practical exercise” (31%), “engagement of the physicians and other medical staff” (27%) and “personal initiative” (23%) resulted in higher subjective fulfilment.

**Conclusions: **The self-chosen LOs reflect the needs of students for which the clinical teacher should be prepared. Considering these findings, it seems possible to close practical training gaps. We support the consideration of establishing curricular anchored self-directed learning in clinical clerkships. Further empirical studies would be beneficial in revealing its positive effects on the learning progress.

## Zusammenfassung

**Hintergrund: **Das höchste Ziel medizinischer Praktika ist es, die Studierenden auf ihren ersten Arbeitstag vorzubereiten. Obwohl die Lehre von klinischer Kompetenz von zentraler Bedeutung ist, bieten klinische Praktika oftmals die letzte Gelegenheit, Wissenslücken mit der Hilfe eines Lehrers aus dem klinischen Bereich zu schließen. Selbstgesteuertes Lernen erweist sich als ein dynamisches Forschungsgebiet in der medizinischen Ausbildung, und dennoch erfolgt seine curriculare Implementierung nur selten. Die vorliegende Studie konzentriert sich unter Anwendung des Konzepts des selbstgesteuerten Lernens auf die allgemeine Bedarfsanalyse klinischer Praktika.

**Methoden: **Die Studie umfasste eine Lernerfahrung an der Ludwig-Maximilians-Universität (LMU) München. Medizinstudenten (n=1446, 59% weiblich) aus dem zweiten klinischen Jahr wurden angeleitet, Lernziele (LZ) mit Hilfe von Doran`s SMART Kriterien zu spezifizieren. Vor Aufnahme des Pflichtpraktikums sollten sie abschätzen, wie wahrscheinlich sie es hielten, diese LZ zu erfüllen. Eine Woche später schätzten sie in einem zweiten Fragebogen ihre tatsächliche und subjektive Zielerreichung. Die erhobenen Daten wurden mit Bezug auf den deutschen Nationalen Kompetenzbasierten Lernzielkatalog Medizin (NKLM) codiert und qualitativ untersucht. Weiterhin wurden bestimmende Faktoren für die Zielerreichung erhoben, und binär codiert (Hindernis vs. Wegbereiter). Sofern angemessen, fand die Methode der univariaten Datenanalyse Anwendung.

**Ergebnisse: **Das Erreichen von „Klinisch-praktische Fertigkeiten“ (29%), „Diagnostische Verfahren“ (21%) und „Ärztliche Gesprächsführung“ (13%) waren die am häufigsten angeführten LZ. Im Laufe der Woche nahm die Zuversichtlichkeit in die Zielerreichung ab. Letztere wurde höher bewertet sofern es reichlich (vs. wenig) Möglichkeiten gab für „Praxisübung“ (31%), „Engagement der Ärzte und des medizinischen Personals“ (27%) und „Eigeninitiative“ (23%).

**Schlussfolgerungen: **Die selbstgewählten LZ sind ein Spiegel des Bedarfs von Studierenden, auf welchen die Lehrer vorbereitet sein sollten. Unter Berücksichtigung dieser Ergebnisse können Lücken in der praktischen Ausbildung möglicherweise geschlossen werden. Wir unterstützen die Überlegung, selbstgesteuertes Lernen in klinischen Praktika curricular zu verankern. Mit Hilfe weiterer empirischer Studien sollten die positiven Effekte auf den Lernfortschritt eindeutig herausgestellt werden.

## Background

The development of academic curricula is important to improve medical education. Curricular models set a clear structure how a planned educational experience best contributes to the learning progress [1]. Aside from other methods, such as seminars or simulation training, clinical clerkships are one of the teaching methods that convey the knowledge to medical students which they need for later for practice. Thereby, a consistent binding set in form of a medical curriculum for clinical clerkships enhances transparency for students, but also for teachers [2]. The measure for the quality of the clinical clerkship is how much it helps the medical students to gain working experience and acquire practical skills [3]. This is crucial, as even young medical professionals have difficulties putting theory into practice [4]. However, practical skills can only develop and be exercised if there is consolidated understanding and knowledge in the field. Therefore, and above all, the aim of clinical clerkships is knowledge growth by the means of problem-based learning [5], [6], [7]. Knowledge per se encloses several different aspects in medical sciences: conceptual knowledge, strategic knowledge, conditional knowledge and procedural knowledge are differentiated and map a spectrum from passive observation to performance [8], [9]. Higher level of knowledge not only correlates with progressed learning but also deeper professional competence [10]. What level of competence (see also Millers pyramid; i.e. knows, knows how, shows how, and do [11]) a student is expected to demonstrate after going through such a clinical clerkship is a relevant content in curriculum development. 

Apart from the mentioned structured education, the second quality that is key to medical skill development in undergraduates is self-directed learning [5], [12]. Its two most prominent characteristics comprise the individual drive to learn independently and intrinsic motivation. As a state of mind, it has been scrutinized in the literature multiple times [13], [14]. Although there are reasons to believe that this non-practical competence may help medical students adapt to the challenges in the clinical environment [15], [16], its implementation to a medical curriculum is rare [17]. One crucial factor for this might be the lack of any general template to follow when designing novel approaches for self-directed learning. Overall, the concept of self-directed learning complies with lifelong learning. Conveying its importance to medical students within limited time (e.g. one week clinical clerkship) is one major obstacle, but student buy-in can be enhanced by fully describing the purpose of self-directed learning to them [18]. 

In this context, the aim of the current study was the critical analysis of medical students’ needs entering a one-week clinical clerkship. In particular, we wanted to find out if the definition of learning objectives by medical students themselves leads to a subjective learning progress. This approach complies with the concept of self-directed learning and might be an alternative solution to the structured approaches to improve learning progress, e.g. by given checklists for documentation.

More specifically, we wanted to answer the following research questions:

1. What are the self-chosen learning objectives (LOs) and the competency levels (CLs) specified for the clinical clerkship?
2. Which of the self-chosen LOs and CLs specified could medical students actually fulfil during their clinical clerkship?
3. What are the barriers and enablers to medical students that affect whether the LOs can be achieved or not?

To answer these research questions we conducted a study among medical students who have successfully passed their theoretical and practical exams in internal medicine facing their obligatory one-week internal medicine clerkship. 

## Methods

### Setting and sample

**Figure 1:**
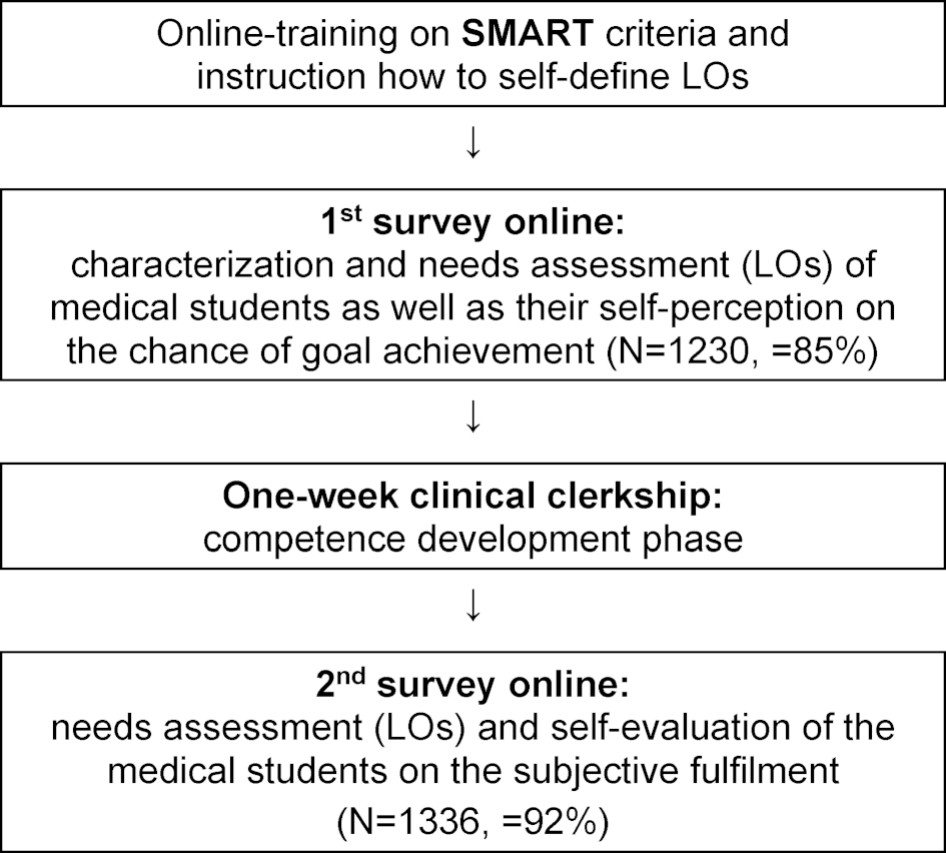
Overview of the study

The study was conducted from April 2016 to March 2019 at Ludwig-Maximilians-Universität (LMU) Munich. During this period, no curricular changes nor substantial teaching staff replacements occurred. Participants in their second clinical year first received a short training on how to specify individual LOs. This training was built on the prior knowledge of the medical students to handle with LOs supported by a link to further documents they already had studied within finished e-learning modules. In order to raise awareness for their individual knowledge gaps in their pre-defined field of focus a specific self-assessment file was made available, too. After that, the students completed a double survey online, one before the one-week internal medicine clinical clerkship and the second after. In total, 1446 medical students (59.1% female) filled in the survey online, for which the request was sent out via e-mail. The participation in the survey was told to be obligatory for successful completion of the course. Its rate of return was 85% and 92% within the first and second questionnaire. In both surveys, they specified at least three LOs. The instructions on how to set the LOs comprised Doran’s SMART criteria. In fact, it uses the meaning of “specific, measurable, achievable, realistic and time-related goals” and helps to phrase goals in a uniform manner [19]. Once the LOs were specified, the medical students also had to rate the probability of their achievement. On day one of the clinical clerkship, the opportunity was given to discuss the individual and structured LOs with his or her supervisor. The quality of the LOs (e.g. too easy or too difficult) was not judged but individual support for goal achievement was made possible. This was followed by the practical competence development phase, after which the medical students rated the actual subjective fulfilment of their LOs. The difference between those ratings (before vs. after) was understood as subjective learning progress. Self-assessed reasons for the success or failure on goal achievement during the clerkship were collected, too. Figure 1 (Fig. 1) shows the course of the study with training on SMART criteria, a preliminary and closing survey online, and the educational experience in between realized by a one-week clinical clerkship. 

#### Coding scheme

A coding scheme was established following the German National Catalogue of Competence-Based Learning Objectives for Undergraduate Medical Education (NKLM). This document gives clarity what knowledge and which abilities a newly licensed physician should have exactly [20]. In order to determine LO categorization, an inductive approach has been selected: following Mayring’s recommendations [21], a relevant sample length of questionnaires – approximately 20% of the total material – was analysed for re-occurring student statements. Based on this analysis, a best-match approach to NKLM was applied resulting in below mentioned categories. Once established the induction step was carried out: application of the scheme to the whole sample set. The latter was possible without any deviations (i.e. no additional categories required, neither single samples). Consequently, the selected category subset was identified to be complete for our problem, while its intra-orthogonality was a priori guaranteed by restricting ourselves to NKLM theory. The final profile of the eight different LO categories comprised as follows:

1. clinically practical abilities
2. diagnostic methods
3. therapeutic principles and medication
4. reasons for encounter
5. professional communication
6. working routine on the ward
7. exam preparation
8. others

By definition, the NKLM describes the learning depth by competency levels (CLs). Within the catalogue “conceptual knowledge”, “procedural knowledge” and “professional practice” are differentiated and follow an ascending order of professional independence. As we did not explicitly ask the medical students to specify CLs, categorisation of each single statement was performed on deductive application of the NKLM’s category set. In order to solve our problem, e.g. capturing all students’ statements, this three CL set was completed by a forth category (“others”) serving as a pool for not further specified LOs. The definition used in the coding scheme is illustrated in table 1 (Tab. 1). 

A final item in the questionnaire asked for the medical students’ explanations for what reasons they had reached (vs. missed) their self-chosen LOs. The received bouquet of arguments was sufficiently comprehensive on student level. However, it cannot be related to a single LO as it depicts the review of the entire one-week clinical clerkship. The collected statements were analysed by process-based category formation (cf. methodology above). As the learning progress is a result of the interaction between students, teachers and the learning environment (e.g. context) [22], [23], best-match to this umbrella three factors categorisation was applied. Subcategory assignment was carried out binary: in case of positive wording, the subcategories were estimated as enablers. Conversely, they were classified as barriers to achieve the self-chosen LOs if the wording was negatively formulated. 

#### Data analysis

Data analysis was performed using the Statistical Package for the Social Sciences (SPSS 25.0, SPSS Inc., Chicago, IL). All statements were discussed jointly by two of the authors and assigned to the determined LOs categories and the operationalized definitions of CL described above. Data of 1446 participants were evaluated. If different LO categories or different CL categories took place at the same time, one response could be coded as more than one category (exemplary quotation that involved simultaneously assignment to category (1) and (2): *“I´d like to derive and interpret ECG”)*. By this, the total number for analysis was 10.754 cases. For examples see table 1 (Tab. 1).

The distribution of the CLs in combination with the self-chosen LOs was examined by the means of cross tables. Chi-squared tests were applied to verify the relationship of the LOs or CLs and participant variables like gender. Differences in the achievement of the self-chosen LOs and CLs were checked by descriptive analysis and comparative averaging. The medical students’ self-evaluation on the achievement of the self-chosen LOs was established in a binary form (enabler or barrier) for univariate analysis. A predefined alpha level set at *p*<.05 was used for all tests of significance. When the data was used multiple times for comparisons we Bonferroni corrected for alpha error accumulation and report results as significant accordingly. 

## Results

**Figure 2:**
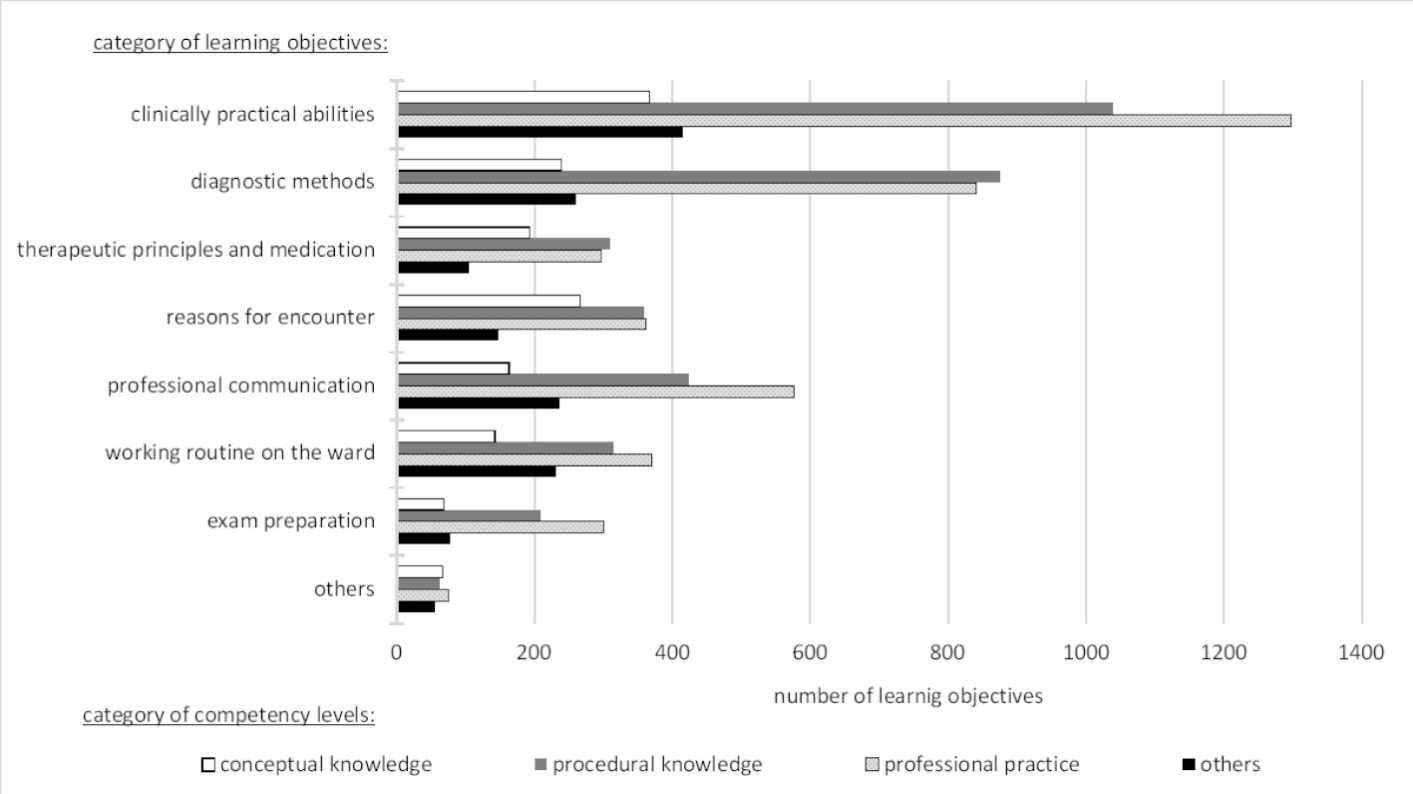
Comparison of the distribution of CLs in combination with self-chosen LOs, featured by absolute frequencies.

The material presented a wide distribution of the self-chosen LOs, each with different CLs. Overall, “clinically practical abilities” (29%), “diagnostic methods” (21%) and “professional communication” (13%) were the LO categories most often mentioned. All LOs contained the whole spectrum of CL categories. The medical students mainly specified the CLs “professional practice” (38%) and “procedural knowledge” (33%). “Conceptual knowledge” as well as “others” were mentioned less frequently (14% each). Figure 2 (Fig. 2) shows how often each LO category contains the four CL categories. “Professional practice” was the leading CL within the LO category “exam preparation” (46%), the CL “procedural knowledge” was connected highest to the LO category “diagnostic methods” (40%), and the CL “conceptual knowledge” appeared primarily within the LO category “reasons for encounter” (23%). The CL “others” predominantly occurred within “working routine on the ward” (22%). Chi-squared tests revealed that the choice of LOs is gender-dependent (Χ^2^(8)=37.2, *p*=.00). While female students strive for “exam preparation”, male students prioritize “working routine on the ward”. Differences between the genders in distributions over the other categories were not significant (*p*-values>.9).

In order to get more insight into the identified needs of the medical students, we investigated which of the self-chosen LOs and concomitant CLs they could actually fulfil during their clinical clerkship. Thus, average self-assessed achievement of each LO was calculated and compared to the average self-estimated values obtained prior to the one-week working experience. The same method was used to detect changes in the concomitant CL achievement. 

**Figure 3:**
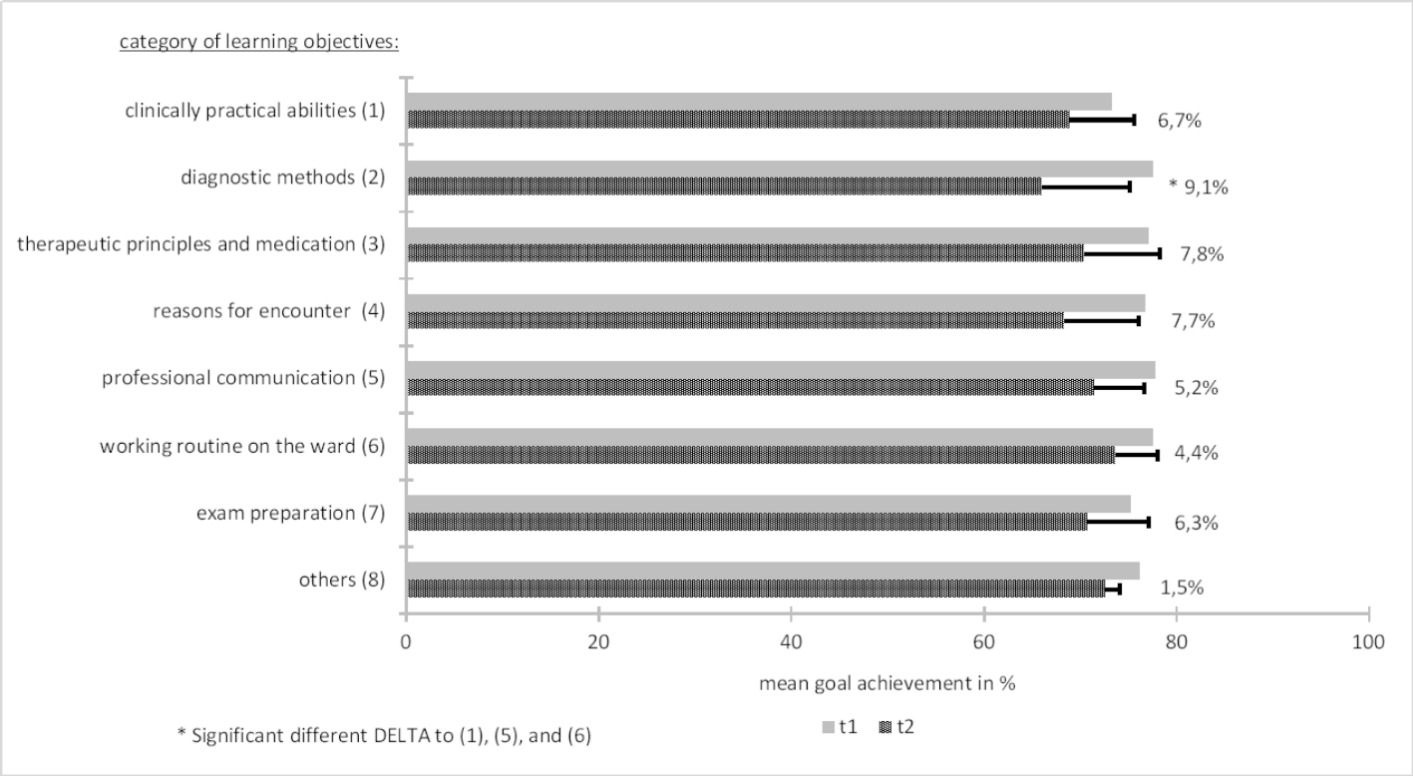
Mean values in percent of the achievement of self-chosen LOs before (=t1) and after (=t2) the one-week clinical clerkship.

At the beginning of the one-week clinical clerkship, participants had an optimistic view towards the fulfilment of their self-chosen LOs. Mean estimates ranged between a 73% and 78% probability. Over the week, actual accomplishment declined to 66%-74%. Apart from “diagnostic methods”, all losses in the achievement of the self-chosen LOs were statistically equal (*p*-values>.9). Figure 3 (Fig. 3) depicts more detailed information on the negative differences. 

**Figure 4:**
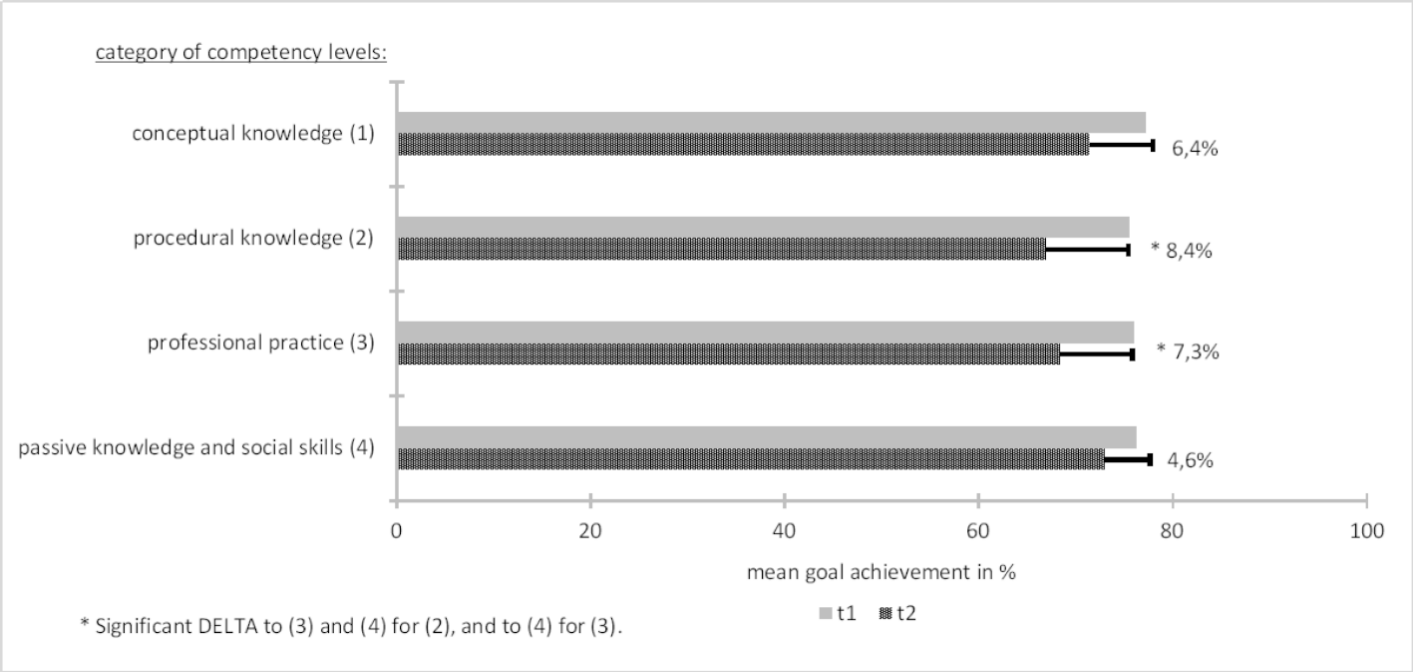
Mean values in percent of the achievement of identified CLs on self-chosen LOs before (=t1) and after (=t2) the one-week clinical clerkship.

A similar pattern occurred for the specified CLs. In the first survey, the collected data showed a 76%-77% probability, and the actual fulfilment decreased to 67%-73%. Figure 4 (Fig. 4) shows that statistically different alterations appeared for “procedural knowledge” and “professional practice”. 

The qualitative content analysis on the barriers and enablers of LO achievement provides more clarity which sets of subcategories in the dimensions “student”, “teacher” and “context” are relevant to our problem. 73.5% (n=1062) of the medical students completed this question. Each given argument was scrutinized for either of two functions: if positively worded, it was classified as enabler, or, if negatively worded, as barrier. Table 2 (Tab. 2) provides information on the identified subcategories. 

**Figure 5:**
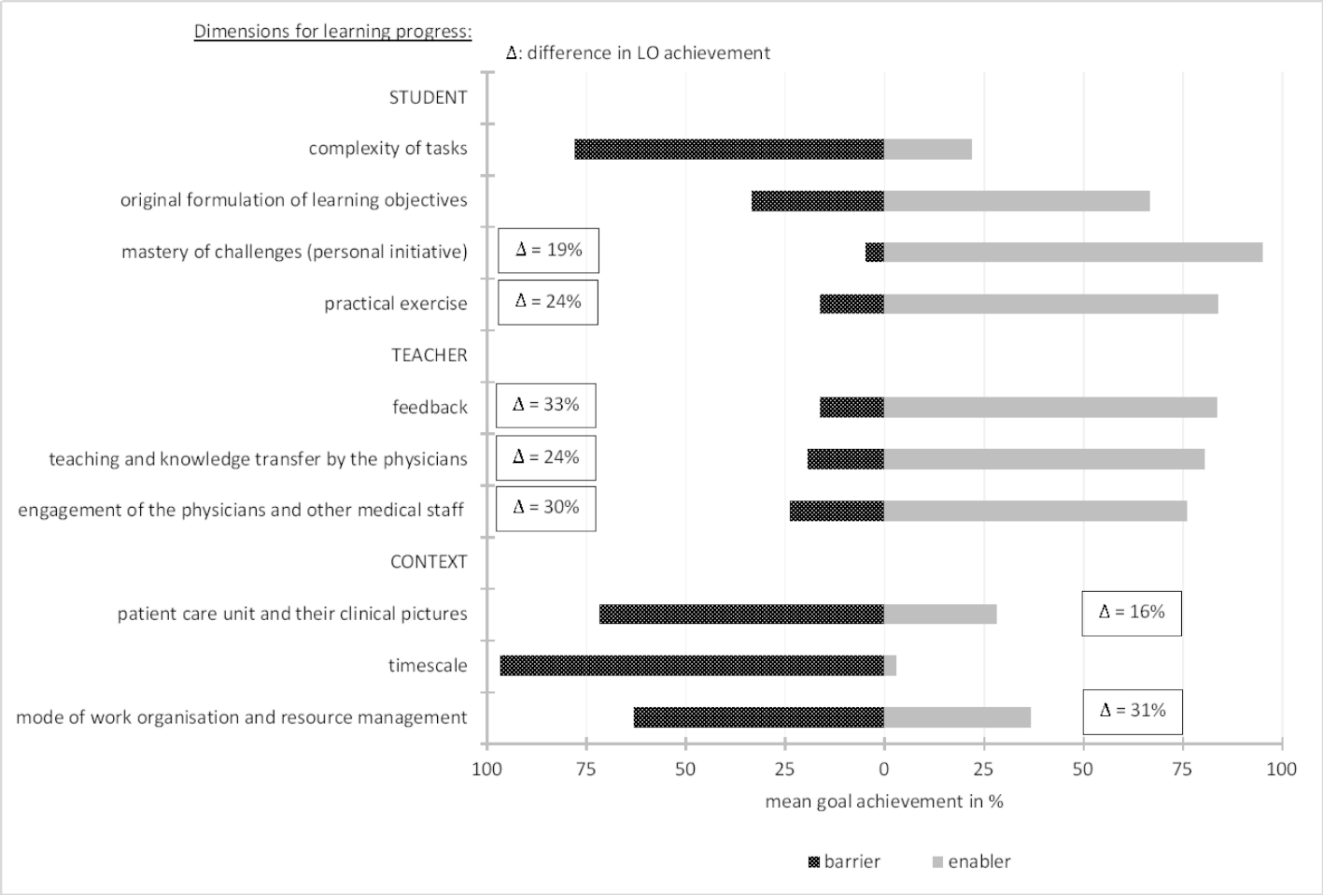
Relative frequencies of barriers and enablers to the achievement of self-chosen LOs. Note: each bar is 100 percent (black part plus grey part). Boxes contain the loss in achievement comparing both conditions (barrier versus enabler). Note: significant delta values are displayed, only.

Figure 5 (Fig. 5) differentiates to which proportion the medical students specified these factors as barriers or enablers. Aside this information, those factors significantly affecting goal achievement of students are highlighted. Details on comprising content of the boxes follow in the next paragraph. 

“Practical exercise” was specified by 31% of the medical students as the cause of learning progress (see table 2 (Tab. 2)). While 16% of this group complained about insufficient practical experience during the clinical clerkship, 84% considered the opposite as true. The latter group achieved the self-chosen LO by 72%, whereas poor practical experience resulted in 48% LO achievement (difference 24%). 

Impact of the “engagement of the physicians and other medical staff” on the achievement of the LOs was mentioned by 27% of the participants. 24% from this group felt hampered by missing clinicians’ commitment, and 76% stated they felt supported to achieve their self-chosen LOs when they perceived engagement. This resulted in 30% better goal achievement. “Personal initiative” to master the upcoming challenges was indicated with a 23% frequency. Thereby, 5% admitted to an own deficit (= barrier), whereas 95% recognized the added value of being personally proactive. The benefit towards goal achievement with this learning strategy was self-estimated by 19%. Figure 5 (Fig. 5) displays all significant delta values of goal achievement comparing both conditions (barrier versus enabler). 

## Discussion

This study was conducted over a period of three years. Before entering a one-week clinical clerkship, medical students specified different LOs which were empirically coded and analysed. The method to self-specify needs when problem-solving fits in current research and investigates how learning progress might be supported [24]. None of the LOs on its own is a guarantee for sufficient working experience but the results of the needs assessment provide a more comprehensive picture. Referring to the NKLM, we could demonstrate that the medical students require competences of all sections implemented in the catalogue. Most often they specified LOs in the field of medical knowledge, clinical skills and professional attitudes, which builds one block in the NKLM. The acquisition of “clinically practical abilities”, “diagnostic methods” and “therapeutic principles and medication” is of special interest to the medical students. On this condition we might make two assumptions:

1. the medical students know their own deficits when facing the clinical clerkship and try their best to eliminate their knowledge gaps
2. the medical students specify especially those LOs for which they anticipate good performance in knowledge-based and clinical assessments

We suppose that the specified LOs are a true representation of the needs that medical students have as they were novices in the clinical practice. The transition to the clinical part requires adjustments in the way of learning and many studies have shown that students do not adapt easily [6], [25]. Learning strategies that worked well in preclinical years are likely to be to the detriment of skill development during the rotations in the clinical environment [26], [27]. By means of, for instance self-directed learning, medical education could facilitate the process of adaption. 

As the third most common of all LOs, the medical students specified the category “professional communication”. This outcome indicates that there is a need to empathize with the physician roles. However, it appears that medical curricula verbalize this competence only informally. So far, some researchers have questioned the so called “hidden” parts of medical curricula [28], [29], [30]. Our results confirm the prior importance of specific role education and training as key enabler for fulfilment of the role as a physician. As a matter of fact, it should be emphasized, such training cannot be approached in generalized manner (viz. a one-fits-all approach), but requires individual adaption to a certain student’s strength/weaknesses.

Regarding the specified CLs, the high priority of “professional practice” and “procedural knowledge” is evident. This priority setting indicates the importance and notable challenge in gaining autonomy in medical practice and execution (the latter as THE major generic aspect required in the role training; refer to the prior paragraph). This observation cannot be related to a single reason, yet it should be regarded as a multi-root cause consequence, as follows:

1. late specialization, i.e. the multitude for mandatory courses in the curriculum hampers students` self-directed learning skills [31],
2. ratio practical/theoretical education not appropriately balanced,
3. insufficient implementation/integration of medical quality control circles, with activities following the Deming cycle comprising the iterative for steps of plan-do-study-act (PDSA cycle) [32],
4. not all aspects of self-education/-optimization can be sufficiently emulated in curricular training (even not more or less artificially scenario courses), but require experience collection during years of practice.

Interestingly, the medical students gave high accuracy ratings to the actual fulfilment of their self-chosen LOs (85%-95%) and CLs (89%-96%). While at first glance their self-assessment skills seem well-functioning, this positive self-evaluation also could have other possible causes: the students may have chosen less challenging tasks where they could demonstrate competence even though they might not have learnt anything new (see first paragraph of the discussion). Or, simultaneously, the psychological phenomenon of the so called Barnum effect might have influenced the observed response behaviour which generally occurs if someone is in the faith that something (here: the self-chosen LOs) is tailored specifically to him [33]. To meet these uncertainties and for a valid comparison, a teachers` assessment of the students` performance or – even better – examination results are needed. 

The results of the descriptive analysis of the barriers and enablers to learning progress confirm our above suggested arguments (i – iv). The concrete propositions used by the medical students underline these sweeping statements of what actions should be taken from an educational perspective. While the medical students recognize themselves as an important influencing factor in the achievement of their LOs, they consider the factors of teacher and context as relevant, too. Herein, from a strategic perspective, maintaining consistency is a logistical administrative challenge inherent to all small group teaching [34]. In the one-week clinical clerkship, with a maximum of two students per patient care unit, the implementation of the self-directed learning component depends on a bouquet of variables amongst them major drivers might be

1. student premedical experience,
2. facilitator expertise or
3. facilitator personal preferences.

The presence of a student in his or her practical year who provides careful supervision might be beneficial. To sum up, achieving consistency among the attendees is a point to bear in mind before setting the exact points at which the medical curriculum shall be reformed. Furthermore, it is absolutely necessary to conduct a second survey in which also teachers of the same peer-group of the actual questioned students are interviewed. 

## Conclusion and outlook

Clerkships frequently serve as connection between acquired clinical knowledge and professional performance at the work setting. In this study, we used the concept of self-directed learning to enable medical students to close knowledge gaps. As this was a qualitative study, properties of the LOs like specificity and difficulty could not be stipulated. However, close proximity and self-evaluation were warranted. According to the concept of self-direction, priority setting of goals (here: LOs) inculcates responsibility in learners to fulfil tasks [14]. At best, it triggers supplementary key processes which many students lack like time management, learning strategies, self-attributions, seeking help and information, and important self-motivational beliefs, such as self-efficacy and intrinsic task interest. On this basis, we now need a controlled study that shows the difference in the achievement of LOs depending on given versus self-chosen LOs. A comparison between self-evaluation and exam results will eliminate bias caused by psychological phenomenon. 

## Abbreviations

- NKLM: German National Catalogue of Competence-Based Learning Objectives for Undergraduate Medical Education
- LMU: Ludwig-Maximilians-Universität
- LO: learning objective
- CL: competency level

## Ethics approval

All analysed data was part of the routine course assessment. The data collection and analysis were completely anonymous. According to the ethical committee of the University Hospital Munich formal consent by the students was not necessary.

## Authors’ contributions

**NR** served as responsible person for analysis and interpretation of the data, including statistics, and the drafting and finalisation of the manuscript. **CL** and **LB **substantially contributed to the study conception and design, the collection and interpretation of the data and the revision of the paper. **BL** and **JF** revised the final manuscript. **MF **contributed to the study design and final version of the manuscript. **RS **designed the study, contributed to data collection, analysis and interpretation, and the drafting and revision of the paper. All authors read and approved the final manuscript. 

## Competing interests

The authors declare that they have no competing interests. 

## Figures and Tables

**Table 1:**
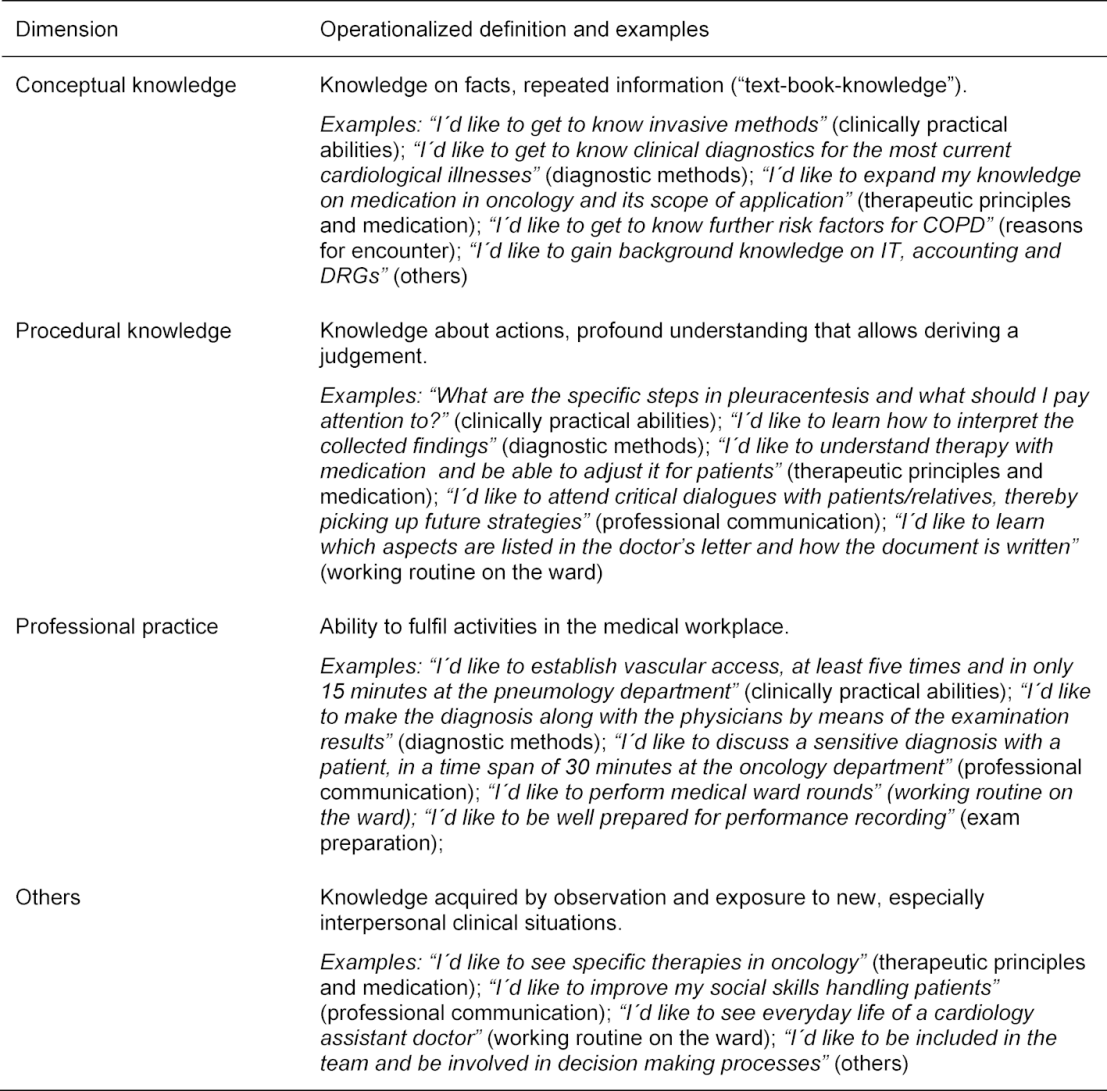
Operationalized definition of the competency level.

**Table 2:**
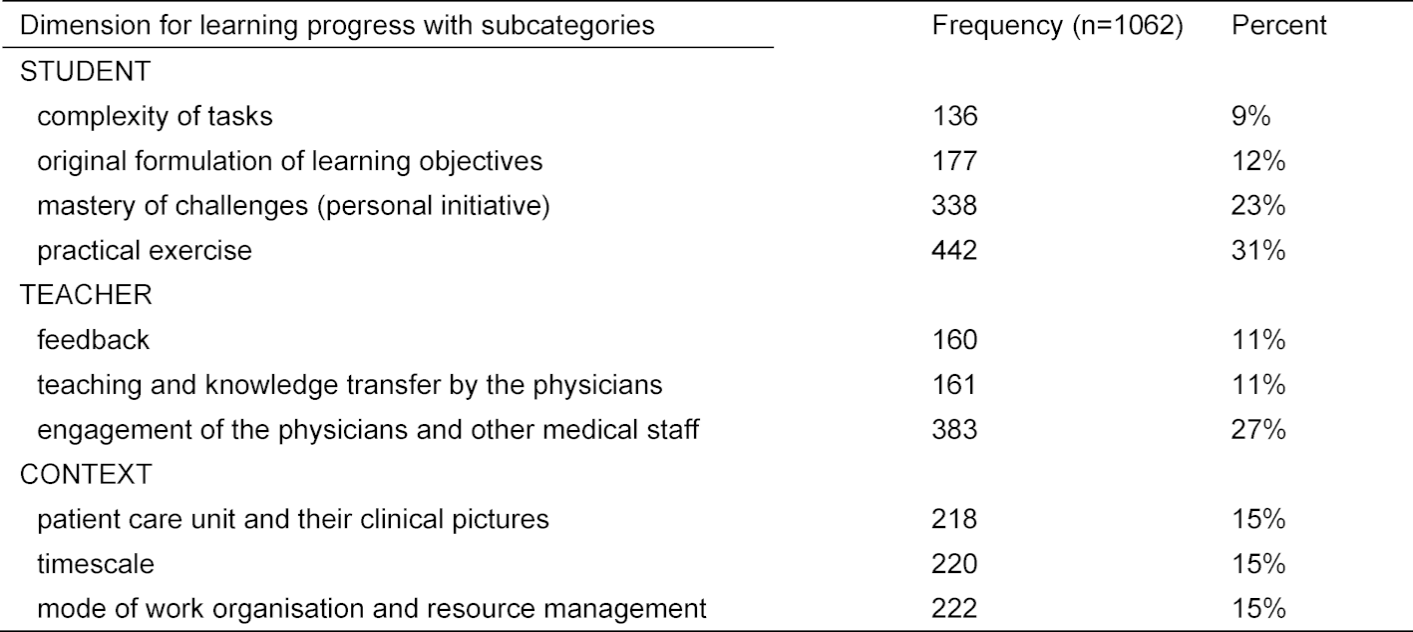
Dimensions for learning progress
